# The Adsorption Capacity of GONs/CMC/Fe_3_O_4_ Magnetic Composite Microspheres and Applications for Purifying Dye Wastewater

**DOI:** 10.3390/ma10010058

**Published:** 2017-01-11

**Authors:** Shenghua Lv, Linlin Zhu, Ying Li, Chunmao Jia, Shiyu Sun

**Affiliations:** 1 College of Bioresources Chemical and Materials Engineering, Shaanxi University of Science and Technology, Xi’an 710021, China; zhull@yahoo.com (L.Z.); Liying0213@yahoo.com (Y.L.); Sunshiyu602@yahoo.com (S.S.); 2 College of Chemistry and Chemical Engineering, Shaanxi University of Science and Technology, Xi’an 710021, China; jiacm1205@yahoo.com

**Keywords:** graphene oxide, carboxymethyl chitosan, magnetic composite microspheres, adsorption

## Abstract

Graphene oxide nanosheets (GONs)/carboxymethyl chitosan (CMC)/Fe_3_O_4_ magnetic composite microspheres (MCMs) were prepared by enclosing Fe_3_O_4_ particles with CMC and GONs in turn. The microstructures of GONs and GONs/CMC/Fe_3_O_4_ MCMs were characterized by FTIR, XRD, TEM, and SEM. The effects of GON content, pH value, and adsorption time on the adsorption capacity of the MCMs were investigated. The results show that the GONs/CMC/Fe_3_O_4_ MCMs have a greater specific surface area and a strong adsorption capacity for dye wastewater. Meanwhile, the adsorption mechanism was investigated, and the results accorded with the pseudo-second-order kinetic model and the Freundlich isotherm model. The search results indicate that GONs/CMC/Fe_3_O_4_ MCMs can be used to purify dye wastewater and has an important potential use in the practical purification of dye wastewater.

## 1. Introduction

Nowadays, the purification technology of wastewater containing dyes and metal ions has become a hot topic worldwide [1,2,3]. The reason is that the dyes and some metal ions such as Cr^3+^, Cr^6+^, Hg^2+^, and Pb^2+^ have high toxicity and are difficult to treat [4]. Several concurrent technologies are being developed for removing dyes and these metal ions from the wastewater: for example, adsorption, flocculation, membrane separation, biological degradation, reverse osmosis, and ion exchange [5,6,7,8,9]. Among these technologies, adsorption is the most suitable method for dye wastewater due to its high efficiency, low cost, and simple process technology [10]. Various traditional adsorbents, such as activated carbon [11], modified clay [12], kaolin [13], peanut hulls [14], and cashew nutshells [15], have been used previously for the removal of dyes from wastewater (Table 1). These adsorbents cannot be separated and recovered for recycling. Therefore, common drawbacks of these adsorbents include their low adsorption efficiency and non-recyclability, which limit their development and application. A fast removal rate and a greater adsorption capacity are required for treating dye wastewater. Therefore, the development of a new adsorbent with a high adsorption capacity and greater adaptability as well as extraordinary regenerative ability is urgent.

In recent years, carboxymethyl chitosan (CMC) has attracted extensive attention because of its potentially high adsorption capacity based on its positive amino groups and biodegradability [16,17,18,19]. However, the good degradation of CMC in water has limited its recyclability. Therefore, the key question is how to keep the best balance between adsorption capacity and recyclability. The usual way to do this is with different kinds of cross-linking agents. Formaldehyde [20], glutaraldehyde [21], epichlorohydrin [22], and adipic acid dihydrazide [23] have been used to form a crosslinking network with CMC to improve its water resistance and volume stability. However, this has not had a remarkable effect. Meanwhile, another problem is that these cross-linking agents themselves have toxicity and may produce new pollution in the use process. In order to significantly improve the adsorption and recyclability of CMC, we introduce here magnetic Fe_3_O_4_ particles and graphene oxide nanosheets (GONs) to form GONs/CMC/Fe_3_O_4_ magnetic composite microspheres (MCMs) to improve its adsorption capacity and regenerative ability. Magnetic Fe_3_O_4_ particles are introduced to easily separate the adsorbents from wastewater through its magnetic polarity. GONs are introduced to increase its adsorption capacity and volume stability by forming crosslinks on the surface of CMC/Fe_3_O_4_ MCMs as well as to improve its specific surface area and to provide more functional groups [24,25]. The research results indicate that GONs have a higher adsorption capacity for many materials such as dyes and the metal ions of Cu^2+^, Hg^2+^, Cr^3+^, Cr^6+^, and Pb^2+^ [26]. In this work, GONs/CMC/Fe_3_O_4_ MCMs were prepared and characterized. The main factors influencing the adsorption capacity of MCMs such as the GON content and pH value as well as the adsorption mechanism were investigated.

## 2. Experimental

### 2.1. Materials and Chemicals

Powdered graphite, concentrated sulfuric acid (H_2_SO_4_, 98%), potassium permanganate (KMnO_4_), hydrogen peroxide (H_2_O_2_, 30%), sodium hydroxide solution (40%), carboxymethyl chitosan (CMC), polyethylene glycol-400 (PEG), Fe_3_O_4_ particles, liquid paraffin, Span-80, Tween-80, glutaraldehyde, absolute ethyl alcohol, and acetone were all purchased from Xi’an Chemical Company Ltd. (Xi’an, China).

### 2.2. Preparation of GONs

**Figure 1:**
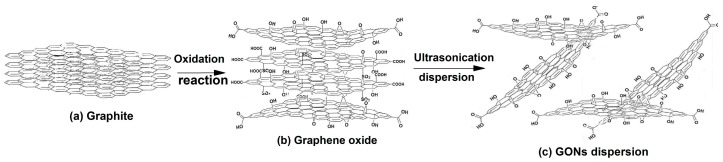
Schematic diagram of preparation process of GONs. (**a**) Compact aggregates of graphite; (**b**) Edge dilation state of graphite in initial oxidation stage; (**c**) Dispersed state of GONs.

Forty grams of H_2_SO_4_ and 1 g of graphite was placed into a 500 mL three-necked flask and kept at less than 5 °C. With continuous stirring, 6 g KMnO_4_ was slowly added to the flask within 1 h. After feeding KMnO_4_, the reaction was sequentially kept at 5 °C for 5 h, and 35 °C for 5 h in turn. Then, 120 g of deionized water was added to the flask and kept at 85 °C for 2 h. Then, about 200 mL of deionized water was added to the flask and kept at 40 °C, and then 7 g of H_2_O_2_ was added dropwise into the flask. At this moment, the color of the solution in the flask became bright yellow. The product was centrifuged and washed with deionized water repeatedly until the pH was about 6–7. Then, about 1000 mL of deionized water was added to the GO, which was then stirred and dispersed by ultrasonication at 1000 W for 1 h. A stable GON dispersion suspension was obtained. The GON concentration was adjusted as about 0.1% by controlling the water content. A schematic diagram of the preparation process of GONs is shown in Figure 1.

### 2.3. Preparation of GONs/CMC/Fe_3_O_4_ MCMs

**Figure 2:**
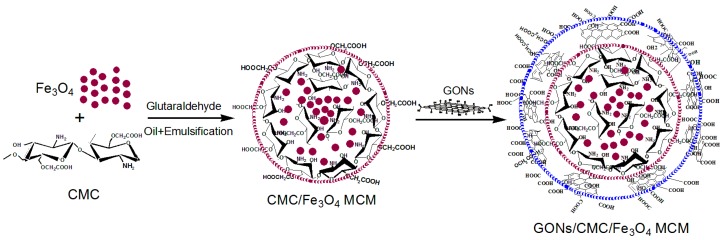
Schematic diagram of preparation process of GONs/CMC/Fe_3_O_4_ MCMs.

We put 3 g of Fe_3_O_4_ particles and 3 g of PEG into 20 g of deionized water to achieve a uniform dispersion, which was then transferred under stirring into an oil phase that contained liquid paraffin, Span-80, and Tween-80. The controlled oil–water ratios were 1:1, 2:1, 3:1, 4:1, 5:1, and 6:1. The amount of oil phase consisted of liquid paraffin, Span-80, and Tween-80 with a mass ratio of 90:10. Then, 30 mL of 20% CMC solution (4 g of CMC solid content) was added and stirred for 30 min, and 2 g of glutaraldehyde was added at 40 °C and stirred for 2 h to prepare the CMC/Fe_3_O_4_ MCMs. Then, different amounts of GONs were added into the CMC/Fe_3_O_4_ MCMs. The GON solid dosages were 0.2 g, 0.4 g, 0.6 g, and 0.8 g. The final product was washed with ethanol and deionized water, and then dried in a vacuum oven at 60 °C. A schematic diagram of the preparation process of GONs/CMC/Fe_3_O_4_ MCMs is shown in Figure 2.

### 2.4. Investigation of Adsorption Capacity of GONs/CMC/Fe_3_O_4_ MCMs

#### 2.4.1. Testing Data Processing Method

In this study, the experimental data were evaluated by the standard deviation. The standard deviation of tested data can be calculated by Equation (1).

(1) $$S=\sqrt{\frac{\sum {\left(x_{i}-\bar{x}\right)}^{2}}{N-1}}$$

where *S* is standard deviation (%), *N* is the total number of samples or all measurement times of each measurement point or sample, *x_i_* is a measurement of a sample, and $\bar{x}$ is the average value of all measurement of a texting point.

#### 2.4.2. Adsorption Capacity and Affect Factors

The adsorption capacity is closely related to the adsorption conditions, such as adsorption time and pH value of the dye wastewater. For the research, the dye wastewater was replaced with a methyl orange aqueous solution at a concentration of 200 mg/L.

The effect of the pH value of the methyl orange solution on capacity was investigated by adjusting the pH values from 3 to 9 with 1 mol·L^−1^ HCl or NaOH dilute solution. The adsorption time was investigated by measuring the adsorption capacity at different times (from initial time to 270 min). The adsorption experiment was performed in an incubator shaker at an oscillation rate of 100 r/min. The methyl orange concentration was determined by a UV–Visible spectrophotometer at 464 nm before and after adsorption. The adsorption capacity was calculated based on Equation (2):

(2) $$Q=\frac{(C_{0}-C_{e})V}{m}$$

where *Q* (mg/g) is the adsorption capacity, *C*_0_ and *C_e_* (mg/L) are the initial and final concentrations of methyl orange solution, respectively, *V* (L) is the volume of the aqueous solutions, and *m* (g) is the mass of the MCMs.

#### 2.4.3. Regenerative Ability

The adsorption experiment was carried out under optimal adsorption process conditions. After the adsorption of dye saturated, the need for regeneration treatment for the MCMs. The regeneration process could be carried out by immersing the saturated MCMs in 6 mol·L^−1^ HCI solution until the adsorption capacity was restored. In fact, the adsorption capacity of MCMs are not likely to return to their original levels, and the decrease in adsorption capacity with regeneration times is inevitable. The cycle times is the regeneration times of the adsorption capacity decreased to about 100 mg/g, which can be used to characterize regenerative ability.

### 2.5. Investigation of Adsorption Mechanism

#### 2.5.1. Adsorption Kinetics

Fifty milligrams of GONs/CMC/Fe_3_O_4_ MCMs were added to 50 mL of 200 mg/L methyl orange solution. The pH of the solution was adjusted to 5, and the adsorption process was conducted at 30 °C under stirring. The concentration of methyl orange was examined every half hour.

#### 2.5.2. Adsorption Isotherms

Fifty milligrams of GONs/CMC/Fe_3_O_4_ MCMs were added to 50 mL of methyl orange solutions with initial concentration from 20 to 200 mg/L. The pH values of methyl orange solutions were adjusted to 5. The concentration of methyl orange was determined after 1 h.

### 2.6. Characterization Methods

The chemical structure of GONs was measured by Fourier-transform infrared spectroscopy (FTIR; EQUINOX-55, Bruker, Ettlingen, Germany) and X-ray photoelectron spectroscopy (XPS; XSAM 800, Kratos, Manchester, UK). The microstructure and the size of GONs were examined via atomic force microscopy (AFM; SPI3800N/SPA400, Seiko, Osaka, Japan) and with a laser particle analyzer (LPA; NANO-ZS90, Zetasizer, Worcestershire, UK). X-ray diffraction (XRD; D/max2200PC, Rigaku, Osaka, Japan) was used to examine the crystalline. The microstructures of the composites were determined with a scanning electron microscope (SEM; S-4800, Hitachi, Tokyo, Japan). The elemental compositions were determined with an energy-dispersive X-ray spectrometer (EDS) (EDAX, Cassatt, SC, USA), which was coupled with the S-4800 SEM. Hysteresis loops were acquired with a vibrating sample magnetometer (VSM, Lake Shore Cryotronics, Westerville, OH, USA). The pore structure of the MCMs was tested using an automatic mercury porosimeter (AMP; Autopore IV9500, Micromeritics, Norcross, GA, USA). The samples were weighed accurately, placed in an expansion joint and sealed, subjected to low pressure (0–30 MPa), reweighed, and then tested at high pressure (30–400 MPa).

## 3. Results and Discussion

### 3.1. Structural Characterization of GONs

**Figure 3:**
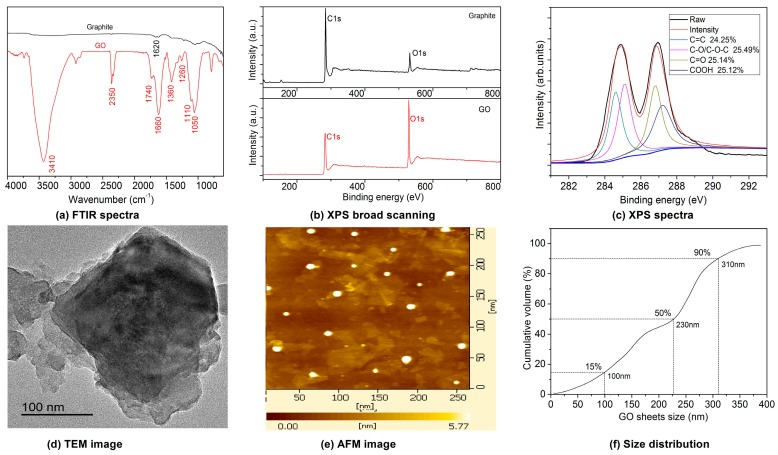
Structural characterization of GONs. (**a**) FTIR spectra; (**b**) XPS broad spectra; (**c**) XPS spectra; (**d**) TEM images; (**e**) AFM images; (**f**) Size distribution.

The structural of GONs were characterized by FTIR, XPS, TEM, AFM, and LPA, and the tested results are shown in Figure 3.

The FTIR spectra of GONs and graphite are shown in Figure 3a. The results indicate that the GONs contain hydroxyl groups (–OH, 3410 cm^−1^), carboxyl groups (COOH, 1740 cm^−1^), carbonyl groups (C=O, 1660 cm^−1^), and ether bonds (–C–O–C–, 1360, 1260, 1100, 1050 cm^−1^), which are not present on the FTIR spectra of graphite. The XPS spectra of GONs are shown in Figure 3b,c, indicating that the graphene oxide has a high oxygen content compared to graphite, and carbon bonds in GO were C=C, C–OH, C–O–C, C=O, and COOH with the proportion of 24.25%, 25.49%, 25.14%, and 25.12%, respectively. The results suggest that GONs contain hydroxyl, epoxy, carbonyl, and carboxyl groups compared with graphite. The TEM images of GONs are shown in Figure 3d, indicating that the GONs are subtransparent and suggesting that GONs are much thinner. AFM images of GONs are shown in Figure 3e. Statistical analysis shows that the average size of the GONs is in the range of 30–420 nm and the maximum thickness is 5.77 nm. The results show that GONs have reached nanometer sheets. Figure 3f shows the size distribution of GONs in an aqueous solution. The results show that the sizes of GONs are in the range of 10–380 nm. The results indicate that 90% of the GONs are smaller than 310 nm and 50% are smaller than 230 nm and 15% are less than 100 nm.

### 3.2. Structural Characterization of GONs/CMC/Fe_3_O_4_ MCMs

**Figure 4:**
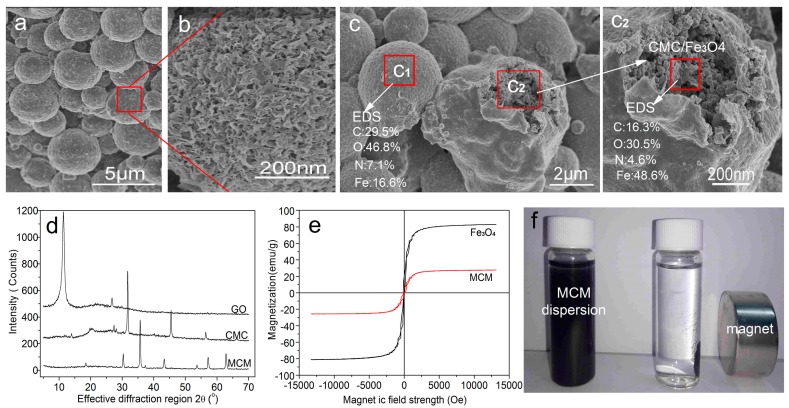
Structural characterization of the MCMs, (**a**) SEM images of the MCMs; (**b**) Microstructure of the MCMs; (**c**) Microstructure and EDS results; (**d**) XRD patterns of the MCMs; (**e**) Magnetic hysteresis loop curves of the MCMs; (**f**) Magnetic separation testing.

The structure of GONs/CMC/Fe_3_O_4_ MCMs was characterized by SEM, XRD, and a magnetic hysteresis loop, and the results are shown in Figure 4.

SEM images of the MCMs prepared with the optimal oil–water ratio of 5:1 are shown in Figure 4a. The results indicate a regular spherical shape and a diameter in the range of 1.3 to 3.3 μm. Figure 4b shows the MCMs with a rough surface formed by the interweaving of fine lamellae. Figure 4C_1_ shows that the surface consists mainly of elemental C and O, suggesting that there are many GONs in the MCM’s surface. Meanwhile, Figure 4C_2_ shows that the internal structure of the MCMs consists mainly of elemental Fe, O, and C. The results indicate that Fe_3_O_4_ particles intertwine with CMC and they are wrapped in shells made from GONs.

The XRD patterns of GONs/CMC/Fe_3_O_4_ MCMs are shown in Figure 4d. The XRD pattern of the GONs has a characteristic peak at 2θ = 11.36°. The characteristic XRD peaks of CMC are at 2θ = 31.74° and 2θ = 45.48°. The XRD peaks of MCMs are at 2θ = 18.46°, 30.34°, 35.7°, 43.32°, 53.72°, 57.22°, and 62.82°, indicating the existence of Fe_3_O_4_ in the MCMs. The results indicate that the XRD peaks of GONs have disappeared in the XRD patterns of MCMs, indicating that the GONs have been distributed uniformly in MCMs because of the intercalation of GONs and CMC.

Figure 4e shows the magnetic hysteresis loop of Fe_3_O_4_ and GONs/CMC/Fe_3_O_4_ MCMs at 30 °C. The results indicate that the saturation magnetizations *M_s_* of Fe_3_O_4_ and GONs/CMC/Fe_3_O_4_ MCMs are 82.5 and 27.53 emu/g, respectively. The saturation magnetization of MCMs is lower than that of Fe_3_O_4_ nanoparticles because the Fe_3_O_4_ particles in the MCMs were surrounded by CMC and GONs. Figure 4f shows that dyes in wastewater adsorbed by MCMs can be separated rapidly from the wastewater in an external magnetic field.

### 3.3. Effect of the Oil–Water Ratio on the Shape of GONs/CMC/Fe_3_O_4_ MCMs

**Figure 5:**
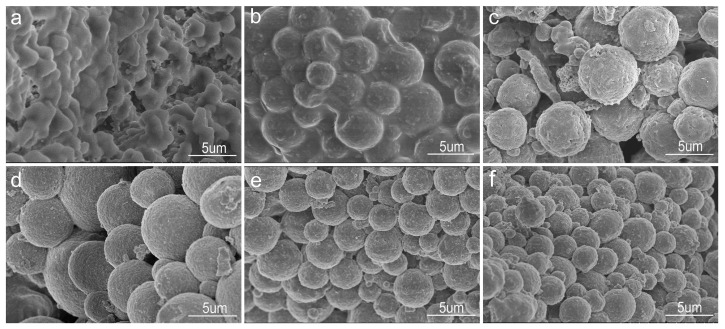
SEM images of GONs/CMC/Fe_3_O_4_ MCMs at different oil–water ratios. (**a**) 1:1; (**b**) 2:1; (**c**) 3:1; (**d**) 4:1; (**e**) 5:1; (**f**) 6:1.

The GONs/CMC/Fe_3_O_4_ MCMs were prepared by mixing 3 g of Fe_3_O_4_, 6 g of CMC, and 0.6 g of GONs at different oil–water ratios, and their SEM images are shown in Figure 5. Figure 5a is a SEM image of GONs/CMC/Fe_3_O_4_ composite with an oil–water ratio of 1:1, indicating that the composite cannot form a spherical shape because the oil–water proportion is unsuitable. Figure 5b shows the SEM images of the GONs/CMC/Fe_3_O_4_ MCMs with an oil–water ratio of 2:1, indicating that the spherical shape of the MCMs had appeared, but that they were bound together. Figure 5c is a SEM image of the MCMs with an oil–water ratio of 3:1, indicating that MCMs with a regular spherical shape had formed. Figure 5d–f are SEM images of MCMs with oil–water ratios of 4:1, 5:1, and 6:1, respectively, indicating that the regular spherical shapes of GONs/CMC/Fe_3_O_4_ MCMs had formed in these oil–water proportions. The results indicate that oil–water ratios of 5:1 and 6:1 are suitable for forming regular MCMs.

### 3.4. Effect of GON Content on Special Surface Area and Adsorption Capacity of GONs/CMC/Fe_3_O_4_ MCMs

Table 2 shows the effects of GON content on the special surface, average pore diameter, and adsorption capacity of GONs/CMC/Fe_3_O_4_ MCMs. The adsorption experiment was carried out using a methyl orange solution with an initial concentration of 200 mg/L at pH 5. The results indicated that doping GONs in the MCMs has a significant effect on the special surface area and adsorption capacity. The results show that the special surface area and adsorption capacity of MCMs obviously increase with an increase in GON dosage from 0.02 g to 0.06 g. When the GON content was 0.06 g, the special surface area and adsorption capacity reached their maximum. At this point, the special surface area and adsorption capacity of MCMs were 126.4 m^2^/g and 164.6 mg/g, respectively. With a further increase in GON content, the special surface area and adsorption capacity remained constant. The adsorption capacity at a GON dosage of 0.06 g increased by 42.3% compared with the control samples. This can be attributed to oversized GONs with a specific surface area and multiple chemical groups, which can significantly improve the adsorption capacity for dyes by increasing the contact area and electrostatic forces between the GONs and the dyes.

### 3.5. Factors Affecting the Adsorption Capacity of GONs/CMC/Fe_3_O_4_ MCMs

The main factors influencing the adsorption capacity of GONs/CMC/Fe_3_O_4_ MCMs for methyl orange are pH value, adsorption time, and the initial concentration of dye in the wastewater.

**Figure 6:**
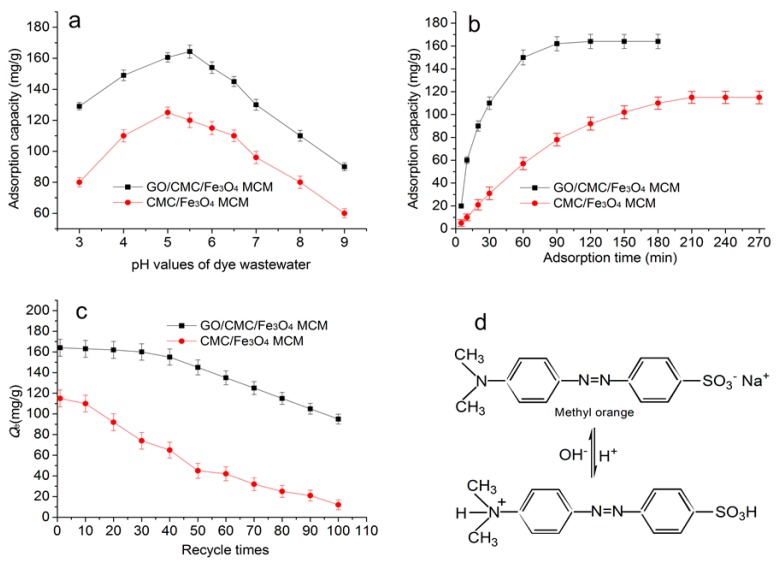
Effects of different factors on the adsorption of the MCMs, (**a**) pH values of dye solution; (**b**) Adsorption time; (**c**) Regenerative ability; (**d**) Methyl orange structure.

Figure 6a shows the effects of the pH values of the dye solution on the adsorption capacity of GONs/CMC/Fe_3_O_4_ MCMs. The results indicate that the adsorption capacity increases with an increase in pH value from 3 to 6; however, with a further increase in pH values from 6 to 9, the adsorption capacity will decrease. The maximal adsorption capacity is 163.2 mg/g when the pH value is 5.5. The reason could be that the amino groups of methyl orange are protonated to –NH_3_^+^ when the pH value is less than 5.5 (Figure 6d), and a greater electrostatic attraction is produced between methyl orange and GONs, resulting in an improved adsorption capacity. When pH values are less than 5.5, the adsorption capacity markedly decreases. The reason is that there are too many cationic particles in dyes that may delay penetration into the deep adsorption layer of the MCMs, because the cationic particles can preferentially combine on the MCM’s surface. When pH values are greater than 7, the dyes will produce many OH^−^ and –SO_3_^2−^ anions, and the adsorption capacity of the MCMs for methyl orange will radically decline.

Figure 6b shows the effect of adsorption time on the adsorption capacity of the MCMs. The results indicate that the adsorption capacity of the GONs/CMC/Fe_3_O_4_ MCMs for dye wastewater achieves equilibrium at 90 min. The equilibrium time for the control sample of CMC/Fe_3_O_4_ MCMs is about 240 min. The results suggest that the adsorption rate of GONs/CMC/Fe_3_O_4_ MCMs for dye wastewater is quick compared with that of CMC/Fe_3_O_4_ MCMs. The equilibrium adsorption capacity is about 164.6 mg/g. The reason is that GONs contribute many chemical groups and a greater special surface area on the MCMs. Therefore, GONs/CMC/Fe_3_O_4_ MCMs for dye wastewater has greater adsorption capacity compared with the traditional adsorbents.

The regenerative ability of the GONs/CMC/Fe_3_O_4_ MCMs was investigated by performing a consecutive adsorption–desorption process. Figure 6c shows the relationship between the recycle times and the adsorption capacity of the GONs/CMC/Fe_3_O_4_ MCMs for methyl orange. It can be seen that the adsorption performance of the GONs/CMC/Fe_3_O_4_ MCMs decreased by 17.7% and 56.2% after 50 and 100 adsorption–regeneration cycles, respectively, while the adsorption performance of the CMC/Fe_3_O_4_ MCMs decreased by 60.9% and 76.5% after 50 and 100 adsorption–regeneration cycles, respectively. This implies that the GONs/CMC/Fe_3_O_4_ MCMs has a greater regeneration capacity than the CMC/Fe_3_O_4_ MCMs and the traditional adsorbents.

### 3.6. Adsorption Mechanism of MCMs

#### 3.6.1. Adsorption Kinetics

To investigate the controlling mechanism of the adsorption processes, pseudo-first-order and pseudo-second-order kinetic models were used to fit the experimental data in this work. The pseudo-first-order kinetic model is generally represented as Equation (3). The pseudo-second-order kinetic model can be expressed as Equation (4).

(3) $$lg(Q_{e}-Q_{t})=lgQ_{e}-\frac{k_{1}}{2.303}t$$

(4) $$\frac{t}{Q_{t}}=\frac{1}{k_{2}Q_{e}^{2}}+\frac{t}{Q_{e}}$$

where, *Q_e_* and *Q_t_* (mg/g) are the adsorption capacity of GONs/CMC/Fe_3_O_4_ MCMs for methyl orange at equilibrium and time *t* (min), respectively. *k*_1_ is the pseudo-first-order rate constant (min^−1^), and *k*_2_ is the pseudo-second-order rate constant (g/(mg·min)).

**Figure 7:**
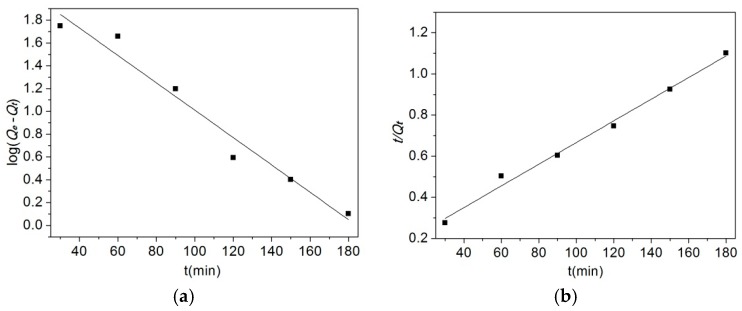
Pseudo-first-order kinetic plots and pseudo-second-order kinetic plots for the adsorption of methyl orange (pH: 5, T: 303 K). (**a**) Pseudo-first order kinetic fitting curve; (**b**) Pseudo-second kinetic fitting curve.

The values of *k* and *Q_e_* (Table 3) were evaluated from pseudo-first-order kinetic plots and pseudo-second-order kinetic plots (Figure 7). As shown in Table 3, according to the correlation coefficients (*R*^2^), the pseudo-second-order equation provided an excellent fit for the experimental data of methyl orange ($R_{2}^{2}$ = 0.9917) compared with the data from the pseudo-first-order equation ($R_{1}^{2}$ = 0.9671). This indicates that the pseudo-second-order adsorption mechanism is predominant.

#### 3.6.2. Adsorption Isotherm

In order to interpret the adsorption experimental data, the most frequently used equations are the Langmuir and Freundlich isotherm equations. The expression of the Langmuir isotherm equation is shown in Equation (5). The expression of the Freundlich isotherm equation is shown in Equation (6).

(5) $$\frac{C_{e}}{Q_{e}}=\frac{C_{e}}{Q_{m}}+\frac{1}{Q_{m}b}$$

(6) $$\mathrm{lg}Q_{e}=\mathrm{lg}k_{F}+\frac{\mathrm{lg}C_{e}}{n}$$

where, *C_e_* is the equilibrium concentration of methyl orange in solution (mg/L), *Q_e_* is the adsorption capacity of methyl orange at equilibrium (mg/g), *Q_m_* is the maximum adsorption capacity (mg/g), *b* is the Langmuir equilibrium constant (L/g), *k_F_* is the Freundlich equilibrium constant (L/g), and *n* is the strength factor.

**Figure 8:**
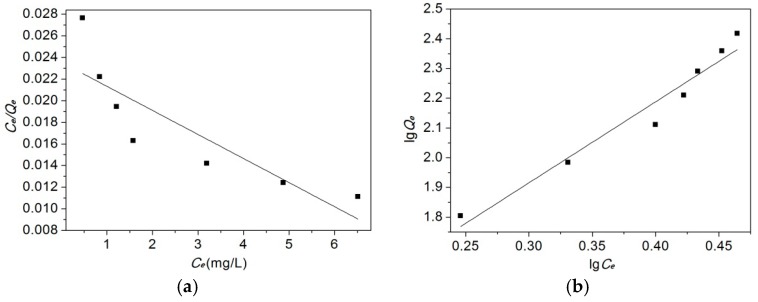
Langmuir isotherm plots and Freundlich isotherm plots for the adsorption of methyl orange. (**a**) Langmuir isotherm adsorption curve; (**b**) Freundlich isotherm adsorption curve.

The obtained parameters of the Langmuir and Freundlich isotherms (Table 4) were evaluated from the Langmuir isotherm plots and Freundlich isotherm plots (Figure 8). It should be noticed that the *R*^2^ values for the Freundlich isotherm models ($R_{1}^{2}$ = 0.955) are higher than those for the Langmuir isotherm models ($R_{2}^{2}$ = 0.7495), which suggests that the Freundlich isotherm fits the experimental data better than the Langmuir isotherm.

### 3.7. Adsorption Capacity of the MCMs for Other Dyes and Metal Ions

Adsorption capacities of the MCMs for dyes and metal ions are shown in Table 5. Nowadays, dyes used in textile, leather, and various other fields have thousands of varieties. According to their chemical structure and dyeing properties, the dyes generally include mainly acid dyes, basic dyes, direct dyes, reactive dyes, and sulfur dyes. These dyes mainly consist of benzene and benzene derivatives, and dyes usually have common chemical groups such as sulfuric groups and carboxyl groups. The adsorption results of Table 5 indicate that GONs/CMC/Fe_3_O_4_ MCMs have excellent adsorption for dyes and some metal ions compared with the modified chitosan in literature (Table 6). We will research the adsorption capacity of the MCMs in another manuscript.

## 4. Conclusions

GONs/CMC/Fe_3_O_4_ MCMs were prepared by inverse emulsion crosslinking to be used as a highly effective adsorbent for purifying dye wastewater. The MCMs had a rough surface of a spherical structure with a large special surface and multiple chemical groups. The MCMs had a higher adsorption capacity for methyl orange and other dye wastewater as well as metal ions due to the introduction of GONs. The results indicate that MCMs can be easily separated and regenerated. The adsorption mechanism was investigated by adsorption kinetics and adsorption isotherms. The research results indicate that the GONs/CMC/Fe_3_O_4_ MCMs have great potential in the purification of dye wastewater.

## Figures and Tables

**Table 1:** Adsorption capacity of traditional adsorbents for dye wastewater.

Ref.	Adsorbents	Adsorbates	Adsorption Conditions	Adsorption Capacity (mg/g)
[11]	Activated carbon	Methylene blue	C* = 600 mg/L, m^✦^ = 0.2 g/L, pH = 7.4, t^✹^ = 35 min	25.3
[12]	Modified clay	Methylene blue	C = 100 mg/L, m = 0.2 g/L, pH = 12, t = 750 min	34.6
[13]	Kaolin	Congo red	C = 150 mg/L, m = 0.5 g/L, pH = 7.5, t = 500 min	5.6
[14]	Peanut hull	Amaranth	C = 50 mg/L, m = 0.5 g/L, pH = 2, t = 15 h	14.9
[15]	Cashew nutshells	Congo red	C = 50 mg/L, m = 0.2 g/L, pH = 2, t = 2 h	5.2

C*: dye wastewater concentration; m^✦^: adsorbent dosage; t^✹^: adsorption time.

**Table 2:** Pore structure of GONs/CMC/Fe_3_O_4_ MCMs.

GONs Content (g)	Specific Surface Area (m^2^/g)	Average Pore Diameter (nm)	Adsorption Capacity (mg/g)
0	46.35	52.5	115.5 ± 0.4
0.02	62.6	31.4	146.3 ± 0.6
0.04	95.2	29.5	156.7 ± 0.7
0.06	126.4	28.3	164.6 ± 0.7
0.08	125.6	28.5	164.4 ± 0.6
0.10	126.4	28.2	164.3 ± 0.8

Compositions of CMC and Fe_3_O_4_ in the GONs/CMC/Fe_3_O_4_ MCMs are 6 g and 3 g, respectively.

**Table 3:** The parameters of the two kinds of dynamic adsorption model.

Pseudo-First-Order Kinetic			Pseudo-Second-Order Kinetic		
*k*_1_ (min^−1^)	$R_{1}^{2}$	*Q’*_e_ (mg/g)	*k*_2_ (L/g)	$R_{2}^{2}$	*Q’_e_* (mg/g)
0.0276	0.9671	162.144	0.000202	0.9917	188.68

Note: *Q*’*_e_* is the equilibrium adsorption from the theoretical calculation.

**Table 4:** The parameters of the two adsorption model.

Langmuir Isotherm			Freundlich Isotherm		
*Q_m_* (mg/g)	*B* (L/g)	$R_{1}^{2}$	*k_F_* (L/g)	*n*	$R_{2}^{2}$
454.55	0.0932	0.7495	12.517	0.367	0.955

**Table 5:** Adsorption capacity of the MCMs for dyes and some metal ions.

Adsorbate	Adsorption Conditions			Adsorption Capacity (mg/g)	
	Temperature (°C)	pH	Adsorption Time (min)	CMC/Fe_3_O_4_ MCMs	GONs/CMC/Fe_3_O_4_ MCMs
Acid red (14720) *	30	5.5	90	115.3 ± 0.5	168.6 ± 0.7
Based black (20470)	30	5.5	90	108.6 ± 0.5	166.5 ± 0.7
Direct black (35255)	30	6.0	90	121.4 ± 0.6	174.2 ± 0.8
Reactive black 8 (2050)	30	6.5	90	118.9 ± 0.5	166.3 ± 0.6
Cr^3+^^✦^	30	6.5	90	21.4 ± 0.4	71.6 ± 0.5
Hg^2+^	30	6.5	90	17.7 ± 0.4	65.3 ± 0.5
Pb^2+^	30	6.5	90	18.6 ± 0.4	58.7 ± 0.4
Cu^2+^	30	6.5	90	15.4 ± 0.4	46.4 ± 0.4

* Color index number, and the initial concentration is 200 mg/L; ^✦^ Initial concentration of the metals ions is 100 mg/L.

**Table 6:** Adsorption capacity of modified chitosan adsorbents.

Refs.	Adsorbents	Adsorbates	Adsorption Capacity (mg/g)	Cycle Times
[27]	GO	Methylene blue	43.5	–
[28]	Natural chitosan membrane	Methylene blue	46.23	<5
[27]	Magnetic chitosan	Methylene blue	60.4	5–10
[27]	GO/Magnetic chitosan composite	Methylene blue	95.16	5–10
[29]	GO/magnetic cyclodextrin–chitosan	Cr^6+^	67.66	5–10
[30]	GO/magnetic chitosan composites	Pb^2+^	76.94	5–10
